# Allocation costs of regeneration: tail regeneration constrains body growth under low food availability in juvenile lizards

**DOI:** 10.1007/s00442-021-05084-6

**Published:** 2021-12-14

**Authors:** Irene Fernández-Rodríguez, Florentino Braña

**Affiliations:** 1 Department of Organisms and Systems Biology (Zoology), University of Oviedo, 33071 Oviedo, Spain; 2 Biodiversity Research Institute (IMIB, UO/CSIC/PA), University of Oviedo, Mieres, Spain

**Keywords:** Autotomy, Resource allocation, Trade-offs, *Podarcis muralis*, Early growth

## Abstract

The balance of energy allocated to development and growth of different body compartments may incur allocation conflicts and can thereby entail physiological and evolutionary consequences. Regeneration after autotomy restores the functionality lost after shedding a body part but requires a strong energy investment that may trade-off with other processes, like reproduction or growth. Caudal autotomy is a widespread antipredator strategy in lizards, but regeneration may provoke decreased growth rates in juveniles that could have subsequent consequences. Here, we assessed the growth of intact and regenerating hatchling wall lizards (*Podarcis muralis*) exposed to different food regimens. Regenerating juveniles presented slightly but significantly lower body growth rates than individuals with intact tails when facing low food availability, but there were no differences when food was supplied ad libitum. Regenerating individuals fed ad libitum increased their ingestion rates compared to intact ones during the period of greatest tail growth, which also reveals a cost of tail regeneration. When resources were scarce, hatchlings invested more in tail regeneration in relation to body growth, rather than delay regeneration to give priority to body growth. We propose that, in juvenile lizards, regeneration could be prioritized even at the expense of body growth to restore the functionality of the lost tail, likely increasing survivorship and the probability to reach reproductive maturity. Our study indicates that food availability is a key factor for the occurrence of trade-offs between regeneration and other growth processes, so that environmental conditions would be determinant for the severity of the costs of regeneration.

## Introduction

Animal life histories exhibit an outstanding diversity, modulated by decisions about the timing of certain events and the allocation of the assimilated energy (Roff 1992; Reznick 2017). Organisms capture and metabolically process energy and materials that they will later assign to various processes, such as body maintenance, somatic growth, reserve accumulation and reproduction (Elliott 1994; van der Meer 2019). Resources are often limited and it is frequent that several traits require energy or materials simultaneously from the same storage, leading to allocation conflicts, so that a great investment in one trait implies fewer resources available for other competing traits. For this reason, trade-offs may have physiological, but also evolutionary consequences, and individuals must balance the proportion (or the timing) of energy allocated to the different traits in a way that maximizes fitness (Stearns 1992; Reznick 2017).

Some animals have the ability to self-mutilate a body part as a reflex response when they are threatened, which is called “autotomy” (Maginnis 2006). Self-mutilation, often followed by the regeneration of the lost parts to restore the organism’s functionality, evolved independently several times in different animal lineages, both invertebrates and vertebrates (Goss 1969; Arnold 1988; Bely and Nyberg 2010; Clause and Capaldi 2006; Lin et al. 2017). Caudal autotomy is a particularly frequent antipredator strategy in lizards, occurring in 13 out of 20 families of saurians (Downes and Shine 2001; McConnachie and Whiting 2003). In addition to its antipredator value, lizards’ tail assumes important functions related to lipid storage (Bellairs and Bryant 1985), communication among conspecifics (Peters et al. 2007) or locomotion (Arnold 1988; Gillis et al. 2013), so that tail loss may negatively impact the performance of relevant ecological functions, thereby affecting fitness (Fox and McCoy 2000; Chapple et al. 2004; Medger et al. 2008; Fleming and Bateman 2012; Hsieh 2016). Caudal regeneration after autotomy seems to restore the functional role of the lost tail in different lizard species (Clause and Capaldi 2006; Zamora-Camacho et al. 2016; see Fernández-Rodríguez and Braña 2020 for *Podarcis muralis*), but re-growing the lost parts requires a substantial input of energy and materials, and this investment may constrain the resources available for other critical whole-organism functions, such as growth or reproduction (Bellairs and Bryant 1985; Maginnis 2006; Bateman and Fleming 2009).

The conflict that arises over the cost of regeneration is likely to be subject to ontogenetic variations (Bateman and Fleming 2009), since other potentially competing, energy demanding processes strongly vary with age. For example, the age of the individual in relation to the onset of reproduction and to its lifespan is expected to have great importance in elucidating allocation conflicts: while adult lizards invest much of the available energy in reproduction and less so in growth, juveniles do not invest in reproduction and have very high growth rates (Andrews 1982; Avery 1970; Steiner and Pfeiffer 2007). Therefore, energy allocated to regeneration in juvenile lizards may diminish the available resources and may constrain body growth (Bernardo and Agosta 2005), even when body size is an important determinant of age at maturity, social rank and mating success in lizards (Vitt et al. 1977). Then, behavioural and physiological changes after tail autotomy are expected to be more extreme in juveniles than in adults (Bateman and Fleming 2009). Besides, stressful environmental conditions, such as low food quality or availability in early stages, may have physiological consequences for the organism, induce accelerated ageing and can have long-term consequences, affecting development, behaviour and physiology later in life (Monaghan 2007; Monaghan et al. 2012). For these reasons, studying the energetic costs of regeneration on body growth and its possible consequences in juvenile individuals is of special interest.

The study of the functional, physiological and ecological implications of regeneration, as well as the possible mechanisms to minimize its costs, is crucial to understand the evolution of autotomy and regeneration in animals. In this context, the aim of the current study was to assess the cost of tail regeneration in early body growth rates in the wall lizard *P. muralis*, comparing growth performance of hatchlings with intact tails with that of regenerating ones. Our experiments were done with newborn lizards that hatched in the laboratory under the same incubation conditions and that had exactly the same age at the beginning of the experiment (2 days, see methods). Therefore, since it is a quite homogeneous sample in which, in addition, there is no interference from any reproductive investment, we consider that it is a very suitable model for the study of the effects of tail regeneration on body growth. As food availability may influence growth rates and the occurrence or intensity of trade-offs (Lawrence 2010; Lynn et al. 2013), we exposed hatchlings with intact or regenerating tails to two different food supply levels, one of which represents a situation of high food availability and the other a situation of food scarcity, likely imposing a conflict of resource allocation without compromising hatchling’s survival and normal development.

## Materials and methods

### Laboratory experiments and measurements

The common wall lizard (*Podarcis muralis*) is a small species in the family Lacertidae (Reptilia: Lacertidae) that occurs in rocky habitats of natural and urban areas of south Europe, from sea level to near 2400 m in elevation (Salvador 2014). Fifty-six gravid females were captured by noose over the course of May 2018 and May 2019 in several close localities of central Asturias (northern Spain), and oviposition occurred in the laboratory between 2 and 20 days after capture in the field. Eggs were incubated individually in covered plastic containers with moistened vermiculite (at a ratio 1:2 of vermiculite to distilled water by weight) at 29 °C, which is the highest temperature at which incubation is the fastest without having negative effects on hatchling phenotypes (Braña and Ji 2000). Hatchlings emerged from the egg after 30–35 days of incubation (mean ± SD: 32.51 ± 0.87 days), and they were weighed (with a digital balance Mettler Toledo AB54 that gave measures to the nearest 0.0001 g) and measured (with a digital caliper Vogel DIN 862 that provided measures to the nearest 0.001 cm) for snout-vent-length (SVL), tail length (TL) and width at the tail base (TW) a few hours after hatching. Hatchlings were sexed by applying a gentle pressure on both sides of the base of the tail, which causes the eversion of hemipenes in males (Harlow 1996; Braña 2008). Sex was confirmed by observing the dimorphic pattern of flank colouration, which is clearly developed in most individuals towards the end of the experimental period. Hatchlings were housed in terraria with water ad libitum containing supplementary vitamins and calcium, and 60 W lamps, to allow behavioural thermoregulation. Hatchlings of each clutch were divided as evenly as possible into two different experimental groups: control (tailed) or experimental (tailless) group; and within each tail group, they were subjected to two different food experiments during one month: food supplied ad libitum, or restricted food. Hatchlings born in 2018 were assigned to the ad libitum treatment, and those born in 2019 were subjected to a restricted food regime; since the trials with both food regimens were conducted in different years, they were considered as two different experiments and analysed separately. Newly hatched lizards were fasted for 2 days to ensure that they had metabolized the remaining residual yolk and were then weighed again. At this point, caudal autotomy was induced to the lizards of the experimental group by firmly grabbing them by the basis of the tail until they detached it. All tailless lizards were left a tail stub of around 0.5 cm (mean ± SD tail stub: 0.482 ± 0.050 cm), corresponding to approximately 7–10 caudal rings/caudal vertebrae.

Hatchlings assigned to the experiment of food ad libitum (*N*_total_ = 89; *N*_tailed_ = 45, *N*_tailless_ = 44) were fed daily mainly with crickets, and they were offered also mealworms once per week to provide a more diverse diet. Food intake was estimated every five days by weighing each lizard before and after eating, and then calculating the weight increase. To estimate food intake, lizards were fasted for 24 h, and they were then fed ad libitum for 30 min. The mass of prey ingested was calculated by weighing each lizard before and after eating.

Hatchlings subjected to food restriction (*N*_total_ = 80; *N*_tailed_ = 41, *N*_tailless_ = 39) were offered one cricket (mean ± SD cricket weight: 0.037 ± 0.006 g) every two days. Once per week, they were offered one mealworm (mean ± SD mealworm weight: 0.021 ± 0.005 g) instead of crickets, to ensure a varied diet. Three days a month (every 10 days) they were fed ad libitum.

Every 10 days, the lizards of all experimental treatments were weighed and measured for SVL, TL, and TW for monitoring their growth during the first month of life. Lizards were always fasted for 24 h before being weighed. To separate the relevant components of total mass of each lizard (i.e., tail and body without tail), we measured (tail length and width) and weighed a sample of shed tails of different sizes and regeneration stages (*N*_intact_ = 34; *N*_regenerated_ = 44) to be able to make estimates of tail mass from tail volume. Linear regressions of tail mass on tail volume had very high coefficients of determination both for intact and for regenerated tails (*R*^2^_intact tails_ = 0.946, *R*^2^_regenerated tails_ = 0.972; *p* < 0.0001 in both cases), and the intercept did not significantly differ from 0 in either case. This indicates a linear isometric relationship between tail mass and tail volume (Packard and Boardman 1987), which allows using the mean ratio mass/volume of the samples of shed tails used in each regressions (one for intact and another for regenerated tails), to estimate tail mass from tail volume. Body mass was then calculated by subtracting the calculated values of tail mass from the total mass.

### Statistical analysis

The assumptions of normality and homoscedasticity were tested by Kolmogorov–Smirnov and Levene tests, respectively. To test for differences in total mass of hatchlings, general linear mixed models were done with tail group and sex as fixed factors and the mother identity as a random factor, for total mass of hatchlings at day 0 (just after inducing tail autotomy to the experimental group) and 30 days after.

To study the investment in body growth vs. tail regeneration, the whole animal was divided into two main compartments: body (without tail) and tail. To test possible differences in longitudinal (SVL) growth, general linear mixed models were done with tail group and sex as fixed factors and the mother as random factor, for SVL at day 0,and for the increase in SVL in 30 days (i.e., SVL at day 30 − SVL at day 0). Besides, a general linear mixed model was done for SVL at days 0, 10, 20 and 30, with tail group, sex and time as fixed factors and mother as random factor. Differences in body mass at day 0 and in the increase in body mass in 30 days were tested by means of general linear mixed models with tail group and sex as fixed factors and mother as random factor.

To study tail growth, we used the increase of tail length and estimated tail mass in 30 days, which adjusted to normality and homogeneity of variances. General linear mixed models with tail group and sex as fixed factors and mother as random factor were done to test for possible differences in growth between intact and regenerated tails. Linear regressions were done separately for tailless and tailed hatchlings of both food experiments to test if tail growth (i.e., the increase of estimated tail mass in 30 days) was related to body growth (i.e., increase of body mass in 30 days). Estimated tail mass increase from day 20 to 30 was tested by a general linear mixed model with tail group as fixed factor, mother as random factor and body mass increase as covariate.

Food intake of animals fed ad libitum was analysed by grouping the six feeding measures taken for each individual in two fortnightly periods of three measures each, considering that these periods correspond to two significant stages of the regeneration process, namely the initial latency phase in which tail regeneration has just started, and the effective regeneration that involves a substantial elongation of the tail. A general linear mixed model with food intake in these two periods as the response variable was carried out to test for possible differences between tailed and tailless animals, and between males and females (tail group, sex and fortnight as fixed factors, mother as random factor).

## Results

### Total growth

**Fig. 1:**
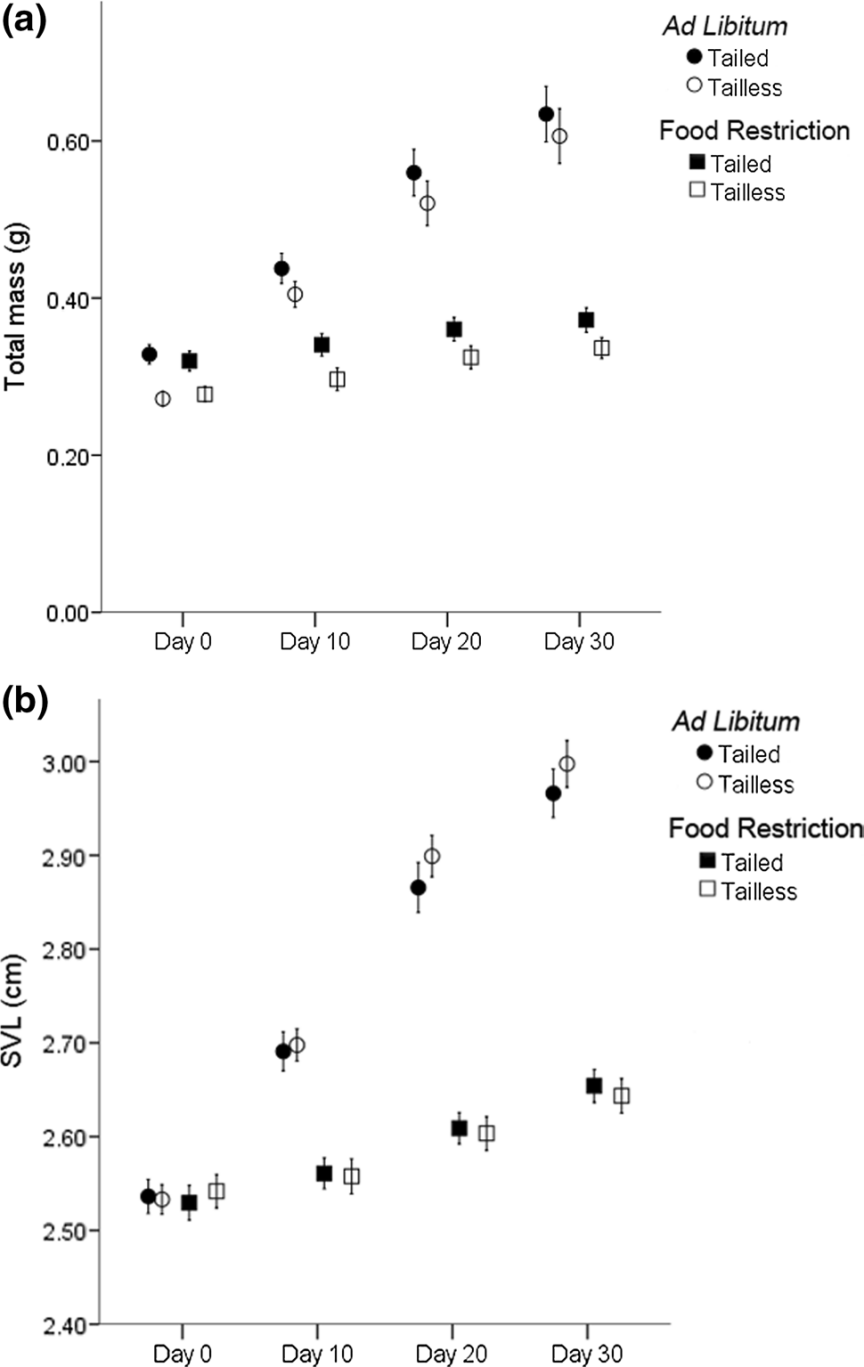
Total mass (body and tail, **a**) and SVL (**b**) in successive time intervals during the first month of life (from day 0 to day 30) of tailed and tailless hatchlings either fed ad libitum or subjected to food restriction. Values are means ± 2SE in A and 1SE in B

As expected, total growth (body and tail) was much more intense in hatchlings on the ad libitum feeding experiment (mean ± SD, Tailed: 0.306 ± 0.099 g; Tailless: 0.334 ± 0.097 g; GLMM with tail group and sex as fixed factors, and mother as a random factor: *F*_1,57_ = 1.596, *p* = 0.217) than in those on the food restriction experiment (mean ± SD Tailed: 0.052 ± 0.033 g; Tailless: 0.059 ± 0.029 g; *F*_1,51_ = 1.527, *p* = 0.222). Obviously, total mass of tailed hatchlings (at day 0) was higher than that of tailless ones that had just lost their tail, and that difference was maintained until the end of the experiment (day 30) both in lizards from the food restriction experiment (Fig. 1A; GLMM: *F*_1,57_ = 36.450, *p* < 0.001) and from the ad libitum experiment (GLMM: *F*_1,57_ = 5.882, *p* = 0.018). Sex was not a significant factor explaining differences of total mass at day 30 between males and females fed ad libitum or with food restriction (GLMMs: *p* > 0.05 in all cases). The interactions between tail group and sex were not significant in any of the former tests.

### Body growth (without tail)

Tailed and tailless lizards of both ad libitum and restricted food experiments did not differ in SVL at hatching, but tailless lizards’ body mass at birth was slightly lower in both experiments (Table 1).

**Table 1:** Descriptive statistics for size and growth of lizards subjected to Ad Libitum (above) and Food Restriction (below) regimens, for female and male tailed and tailless hatchlings

	Tailed			Tailless			Tail group		Sex	
	Females	Males	Total	Females	Males	Total	*F*	*p*	*F*	*p*
*Ad Libitum*										
Initial SVL (cm)	2.580 ± 0.118	2.494 ± 0.109	2.536 ± 0.121	2.543 ± 0.087	2.522 ± 0.123	2.532 ± 0.105	0.408	0.526	**6.738**	**0.012**
SVL increase (cm)	0.418 ± 0.113	0.442 ± 0.194	0.429 ± 0.103	0.469 ± 0.123	0.455 ± 0.105	0.462 ± 0.114	2.194	0.144	0.073	0.788
Init. body mass (g)	0.295 ± 0.028	0.266 ± 0.039	0.280 ± 0.037	0.271 ± 0.025	0.273 ± 0.039	0.272 ± 0.032	**6.337**	**0.015**	1.823	0.182
Body mass incr. (g)	0.258 ± 0.094	0.249 ± 0.069	0.254 ± 0.081	0.267 ± 0.072	0.291 ± 0.086	0.279 ± 0.079	1.862	0.178	0.722	0.399
Tail leng. incr. (cm)	1.698 ± 0.332	1.731 ± 0.273	1.731 ± 0.301	2.123 ± 0.455	2.269 ± 0.491	2.194 ± 0.472	**56.47**	** < 0.001**	0.999	0.322
Tail mass incr. (g)	0.059 ± 0.024	0.045 ± 0.023	0.052 ± 0.024	0.052 ± 0.018	0.057 ± 0.026	0.055 ± 0.022	0.275	0.612	0.359	0.551
Food restriction										
Initial SVL (cm)	2.590 ± 0.097	2.459 ± 0.104	2.529 ± 0.119	2.557 ± 0.129	2.524 ± 0.087	2.542 ± 0.112	0.719	0.400	**20.395**	** < 0.001**
SVL increase (cm)	0.108 ± 0.053	0.144 ± 0.053	0.125 ± 0.055	0.092 ± 0.095	0.114 ± 0.056	0.102 ± 0.051	**4.833**	**0.033**	**6.935**	**0.011**
Init. body mass (g)	0.292 ± 0.031	0.267 ± 0.031	0.280 ± 0.033	0.271 ± 0.027	0.285 ± 0.033	0.278 ± 0.030	**4.510**	**0.039**	0.032	0.858
Body mass incr. (g)	0.038 ± 0.027	0.038 ± 0.029	0.038 ± 0.028	0.042 ± 0.017	0.048 ± 0.035	0.044 ± 0.026	2.015	0.163	0.623	0.434
Tail leng. incr. (cm)	0.958 ± 0.324	1.043 ± 0.309	0.997 ± 0.316	1.003 ± 0.327	0.961 ± 0.369	0.983 ± 0.343	1.048	0.311	0.853	0.360
Tail mass incr. (g)	0.013 ± 0.009	0.016 ± 0.008	0.014 ± 0.009	0.014 ± 0.007	0.015 ± 0.008	0.015 ± 0.007	0.011	0.915	1.247	0.269

Significant values are highlighted in bold

The increases refer to the total increase from day 0 to day 30. General linear mixed models were done with tail group and sex as fixed factors and mother as random factor in all cases

*Init*. Initial, *leng*. Length, *incr*. increase. Values are means ± SD

#### Ad libitum experiment

Growth in length (SVL) was not significantly different for tailless and tailed lizards fed ad libitum, neither after 30 days (Table 1), nor in the successive measurements of body length during one month (GLMM with tail group, sex and time as fixed factors, and mother as a random factor: *F*_1,57_ = 0.054, *p* = 0.817). Regarding body mass, no differences were found in the increase on body mass in 30 days between tailed and tailless hatchlings fed ad libitum (Table 1). Females were significantly longer at birth than males (Table 1) and these differences tended to disappear after 30 days (GLMM: *F*_1,57_ = 2.945, *p* = 0.092), although growth was statistically not significantly greater in males than in females (Table 1). No differences were found between males and females in body mass neither at the beginning of the experiment, nor in the growth in body mass during 30 days (Table 1). The interactions between tail group and sex were not significant in any of the former tests.

#### Food restriction experiment

Regarding the food restriction experiment, the effect of tail group on the successive measurements of body length during one month was marginally significant (GLMM of SVL at days 0, 10, 20 and 30 with tail group, sex and time as fixed factors and mother as a random factor: *F*_1,51_ = 3.552, *p* = 0.065). Besides, there was a significant interaction between tail and time (*F*_1,236_ = 5.574, *p* = 0.019), as tailed lizards were slightly smaller in SVL at the beginning of the experiment but significantly larger at the end, and the differences in SVL between tailed and tailless lizards increased with time (Fig. 1B). In addition, the total increase of SVL in these 30 days was significantly higher in tailed lizards (Table 1). There were no differences in body mass growth after 30 days between tailed and tailless hatchlings (Table 1). Females were significantly longer at birth than males and, although males grew significantly more than females in SVL (Table 1), females remained larger than males in SVL after 30 days (GLMM: *F*_1,51_ = 5.279, *p* = 0.026). No differences were found between males and females in body mass neither at the beginning of the experiment, nor in the growth in body mass during 30 days (Table 1). The interactions between tail group and sex were not significant in any of the former tests.

### Tail growth

Regeneration rate in tailless hatchlings fed ad libitum was much faster than in those subjected to food restriction (mean ± SD tail regenerated in 30 days, ad libitum: 0.055 ± 0.022 g; food restriction: 0.015 ± 0.007 g). There were no significant differences in the increase of estimated tail mass in 30 days between intact (tailed individuals) and regenerated tails (tailless ones) of hatchlings from the ad libitum and food restriction experiments (Table 1). Tail length increase was not different for tailless and tailed lizards in the food restriction experiment but was significantly higher for tailless lizards from the ad libitum experiment (Table 1). No between-sex differences were found in the increase of estimated tail mass or tail length for hatchlings fed ad libitum or with food restriction (Table 1). The interactions between tail group and sex were not significant in any of the former tests.

**Fig. 2:**
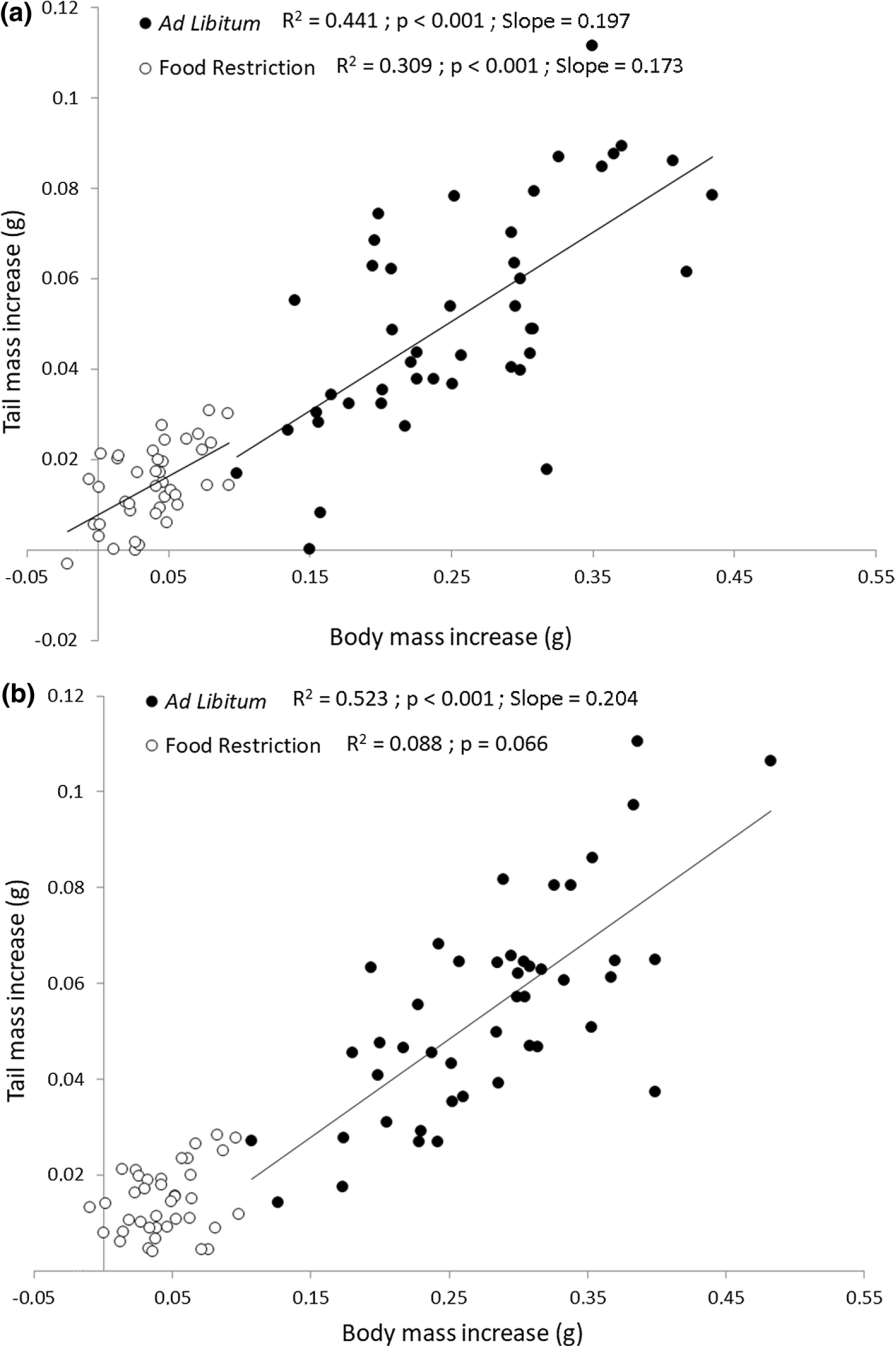
Body growth against tail growth (i.e., estimated increase of mass) in 30 days of **a** tailed and **b** tailless hatchling lizards that were fed ad libitum or underwent a food restriction regime. The data for the two feeding regimes come from two independent experiments

**Fig. 3:**
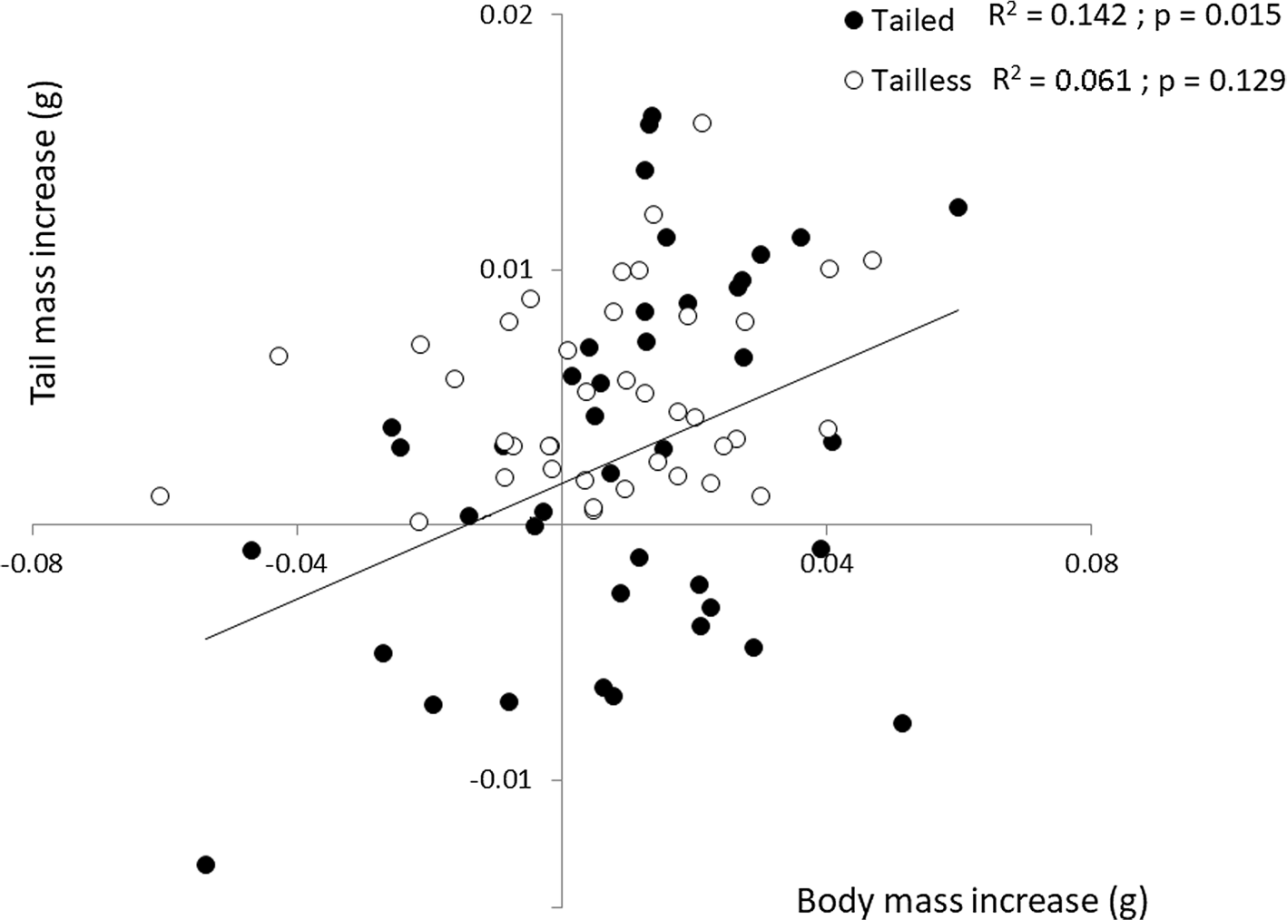
Estimated body growth (tail excluded) against tail growth during the third experimental period (from day 20 to day 30 after hatching) of tailed and tailless hatchlings subjected to food restriction. The regression line corresponds to the relationship for tailed lizards

There was a positive relationship between estimated body and tail mass growth for tailed hatchlings, both for ad libitum and restricted food experiments (Fig. 2A; ad libitum: *R*^2^ = 0.441, slope = 0.197 ± 0.034 (standard error), *p* < 0.001; restricted food: *R*^2^ = 0.309, slope = 0.173 ± 0.041, *p* < 0.001), with similar slopes. Tailless individuals fed ad libitum also showed a positive relationship between these variables, but no significant relationship was found for lizards subjected to food restriction (Fig. 2B; ad libitum: *R*^2^ = 0.523, slope = 0.204 ± 0.029, *p* < 0.001; restricted food: *R*^2^ = 0.088, *p* = 0.066). Besides, estimated tail growth investment of lizards with food restriction was low during the first 10 days (although slightly higher in tailed individuals than in regenerating ones), both in absolute values and in relation to body growth, but estimated tail growth (both for intact and regenerated tails) reached its maximum during the days 10 to 20, being higher in tailed individuals in relation to estimated body growth. Finally, in the third period measured (from day 20 to 30), regenerating individuals invested more in tail growth in relation to body growth than did intact ones (Fig. 3; GLMM with log_10_-transformed estimated tail mass increase from day 20 to 30, with tail group as fixed factor, mother as random factor and log_10_-transformed body mass increase as covariate: *F*_1,23_ = 6.669, *p* = 0.017).

### Food intake

#### Ad libitum experiment

**Fig. 4:**
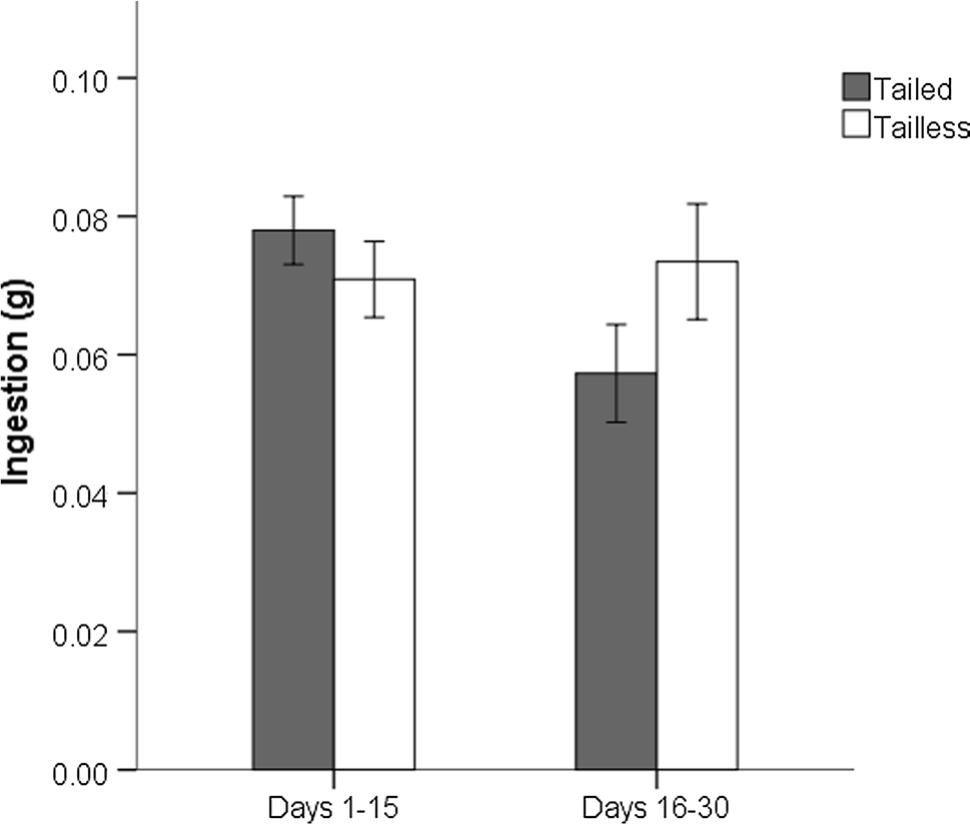
Mass of prey ingested by tailed and tailless hatchlings fed ad libitum, grouped in two fortnightly periods. Values are means ± SE of three measures made to all lizards in each period

There were no significant differences in food intake between tailed and tailless lizards fed ad libitum in the two different fortnights (GLMM with tail group, sex and fortnight as fixed factors, and mother as a random factor: *p* > 0.05). However, there was a significant interaction between periods of the ingestion rate of tailed and tailless individuals (GLMM, interaction between fortnights and tail group: *F*_1,84_ = 4.113, *p* = 0.046): tailless hatchlings increased their ingestion rate relative to tailed ones in the second fortnight (Fig. 4).

#### Food restriction experiment

Ingestion of hatchlings subjected to food restriction was fixed and they ingested a mean of 0.368 ± 0.048 g (mean ± SD) of prey in the whole month. Food intake was homogeneous for tailed and tailless hatchlings and for males and females of the restricted food experiment (GLMM with tail group and sex as fixed factors, and mother as a random factor: *p* > 0.05 in both cases).

## Discussion

Caudal autotomy has been shown to entail significant functional costs in many species of lizards, affecting locomotion, foraging habits, mating success, habitat use and social status (Fox and Rostker 1982; Fox et al. 1981; Bateman and Fleming 2009). Some long-term studies evidenced that these costs can decrease survivorship and thus the overall lifetime fitness of the individual (Fox and McCoy 2000; Lin et al. 2017). However, experimental studies have given less attention to the investment of energy and materials associated to regeneration following autotomy and the potential subsequent costs for growth or reproduction. Theoretical predictions state that regeneration may trade off with other processes, such as reproduction or growth, that occur simultaneously and that have also a high demand on energy and materials (Maginnis 2006), but the consequences of re-growing the tail on body growth remains rather unknown. Our study revealed that tailless (i.e., regenerating) juvenile wall lizards had slightly but significantly lower growth rates in body length than individuals with intact tails when facing situations of low food availability, but there were not such differences when food was supplied ad libitum. Similarly, Lynn et al. (2013) found that juvenile leopard geckos (*Eublepharis macularius*) had reduced body growth rates after autotomy when they had limited food resources. Although the differences in growth (SVL) between tailed and tailless lizards observed in this study might seem minimal and therefore of low biological relevance, these differences could thus probably increase with time if a regime of low food availability is maintained, likely attaining higher biological relevance. Regarding sex-related differences, females were larger in SVL than males at birth in both experiments, and males grew more than females during the experiment with food restriction but did not reach females’ size after 30 days. It is worth noting that no interaction between sex and tail group was significant in any of the measured variables. This model of sexual dimorphism in size at birth (being females longer than males) and initial body growth (higher in males) has been previously reported for this species (Braña and Ji 2000).

In contrast to our results, most of the few studies that have addressed the costs of tail regeneration on body growth in lizards have not found evidence of such costs. Some of these studies were laboratory experiments performed under controlled conditions, but food was supplied ad libitum, which could have masked the possible trade-off between tail regeneration and body growth (Ballinger and Tinkle 1979; Chapple et al. 2004; Goodman 2006; Iraeta et al. 2012; Starostová et al. 2017), as suggested by our on data, since the slopes of body growth against tail growth were almost equivalent for tailed and tailless lizards fed ad libitum. Althoff and Thompson (1994) made similar experiments to ours, subjecting individuals of the side-blotched lizard (*Uta stansburiana*) to different food treatments (low, medium and ad libitum food supply) to avoid overlooking a possible trade-off; however, they did not find differences in growth rates, neither among tailed and tailless lizards, nor between food treatments. This contrasts sharply with the results obtained in our experiment, in which growth rates of lizards fed ad libitum were 3.9 times higher than those of lizards subjected to food restriction. The most plausible explanation for that discrepancy is that the amount of food that Althoff and Thompson (1994) provided to their group with the most severe food restriction was almost twice the amount provided to hatchlings in our restricted food experiment (for lizard species of similar size), so it is likely that that restriction was not enough to impose a major constraint on growth.

With regard to field studies, some of them have reported diminished body growth rates in regenerating lizards, which were generally attributed to possible limitations of food intake during regeneration (Ballinger and Tinkle 1979; Smith 1996; Niewiarowski et al. 1997; Salvador and Veiga 2005). However, other field studies have found no effect of tail regeneration on body growth rates of lizards, and some authors speculated about possible higher ingestion rates of regenerating individuals (Van Sluys 1998; Fox and McCoy 2000; Webb 2006). Environmental conditions, including prey abundance, will likely determine the severity of the costs of regeneration in juveniles under natural conditions.

Hatchlings and juvenile lizards generally exhibit high growth rates (Andrews 1982; Avery 1970) and have therefore a high energy demand, which may even increase in situations of additional requirements, such as tail regeneration. The regeneration process first begins with the recovery and repair of the injury, cell differentiation and blastemal formation (Bellairs and Bryant 1985; Bryant et al. 2002), so that cell proliferation and tail elongation start a few weeks after autotomy (e.g., 4–5 weeks in the leopard gecko *E. macularius,* McLean and Vickaryous 2011, and 1–2 weeks in our hatchlings). There is conflicting evidence of how tail regeneration may affect metabolic rates in lizards. For instance, Starostová et al. (2017) did not find significant differences between control and regenerating lizards in the gecko *Paroedura picta*, whereas Naya et al. (2007) reported a substantial increase (36%) in the standard metabolic rate of *Liolaemus belli*. Our results support the idea that regeneration requires a significant demand of energy, since regenerating individuals grew less (under food restriction) or increased ingestion rates in relation to intact ones (when fed ad libitum), precisely at the time when the effective growth phase of regeneration begins. In return for the advantages of acquiring more resources, juveniles that face increased energy requirements may spend more time foraging, increasing exposure and predation risk (Dial and Fitzpatrick 1981; Fox 1978). Besides, juvenile lizards sometimes face agonistic interactions with adults, being restricted to more limited and often suboptimal territories (Brandl and Völkl 1988). This, together with an impaired locomotion due to tail loss (Medger et al. 2008; Gillis et al. 2009; see Fernández-Rodríguez and Braña 2020 for *Podarcis muralis*), could affect their access to food resources and foraging efficiency.

Our results indicate that body growth and tail regeneration are not positively correlated when resources are scarce (i.e., low food availability), but in these conditions hatchlings seems to invest more energy in tail regeneration in relation to body growth, rather than delay regeneration to give priority to body growth. Contrary to this finding, Vitt et al. (1977) suggested that regeneration should be selected to be slow in long-lived species with high probability of surviving to the next reproductive season, and juveniles should prioritize allocation on body growth over tail regeneration more than adults (but see Tinkle 1967; Lynn et al. 2013). As predation is usually size-related, juvenile lizards are likely to have more potential predators, and thus face a higher predation risk than adults (Blomberg and Shine 2000). As a consequence, tail autotomy is very frequent in juveniles (Chapple et al. 2004), and it is a very important antipredator mechanism, because locomotor performance and other abilities are not yet well developed (Iraeta et al. 2012). As an example of the relevance of this mechanism, juveniles of many lizard species exhibit striking colourations in the tail, which may attract predators’ attention and deflect the attacks from the head or body, hence increasing the chance of survivorship (Cooper and Vitt 1985; Castilla et al. 1999; Pianka and Vitt 2003; Kuriyama et al. 2016). However, lizards are more vulnerable after autotomy, as they have lost one effective defense against predators (Congdon et al. 1974; Wilson 1992; Fox and McCoy 2000; but see Daniels 1983; Ding et al. 2012) and tail loss has been proved to impair locomotor performance (Chapple et al. 2004; Sun et al. 2009; Fernández-Rodríguez and Braña 2020). Regeneration has long-term antipredator value (Tsasi et al. 2009; Lin et al. 2017) and, under this framework, rapid regeneration rates (giving even priority to tail re-growth at the expenses of body growth) would be important for the individuals’ fitness and could have been selected in juveniles of some species (like *P. muralis*) to increase the probabilities of survival until the first reproductive season. In such case, investing in tail regeneration would have immediate benefits (e.g., restoring locomotor capacities, which may improve feeding or diminish predation risk) implying lifetime fitness consequences.

Decreased body growth due to energy allocated to regeneration can delay approaching to the asymptotic size and can even lead to a smaller final size, with important potential consequences for lifetime fitness, as body size can affect metabolic rates, age at sexual maturity, social rank, territory use, fecundity, mating success and survival in lizards (Brownikowsi and Arnold 1999; King et al. 2016; see Peters 1983 for a general account). Besides, fat reserves and body size reached at the beginning of hibernation are important for winter survival of juvenile lizards (Bauwens 1981; Civantos et al. 1999; Iraeta et al. 2012), and some authors have suggested that there might be selective advantages to reach early the minimum body size at maturity (Iraeta et al. 2008). Reduction of growth rate during tail regeneration could even trigger compensatory growth responses in juveniles once regeneration finishes, to reach a minimum body size (Vogel et al. 1986; Dmitriew 2011), although compensatory growth is known to affect physiology later in life (e.g., maintaining high metabolic rates in adulthood; Criscuolo et al. 2008). Finally, although the high metabolic demands during tail regeneration and its impact on juvenile’s body growth could be finally fulfilled or compensated to diminish or avoid the costs of decreased body size (as suggested by our results), those stressful conditions during early life stages may have long-term consequences in adulthood, affecting physiology later in life, or reducing reproductive investment or lifespan (Monaghan 2007; Inness and Metcalfe 2008). Further research on the consequences of regeneration during early life is needed, considering not only immediate and short-term effects (during juvenile stages), but also long-term effects during adulthood, that could affect reproductive output and life-time individual fitness.

To conclude, in general terms, and according to the results of our study and the available literature, regeneration does not impose extremely high additional energy demands, but it may compromise body growth when environmental conditions (food availability) are unfavourable. Our data provided evidence that food shortage has negative consequences for regeneration and body growth, which could be especially critical for hatchlings, as they have narrow range of potential preys and do not have fully developed predatory skills. Besides, tail loss affects locomotor performance of lizards and therefore reduces their efficiency as predators. For these two reasons, it is likely that this scenario of food scarcity may occur in the wild. It seems, therefore, that the availability of food is a determinant for the occurrence of a trade-off between regeneration and other growth processes, which agrees with some experimental studies conducted in other animals with high regenerative capacities, mainly echinoderms (Díaz-Guisado et al. 2006; Barrios et al. 2008; Lawrence 2010; Ramsay et al. 2001). Caudal autotomy and tail regeneration are very common and key antipredator strategies for juvenile lizards, and from our results we propose that tail regeneration in juveniles may be prioritized even at the expenses of body growth, allowing to restore the lost functionality as soon as possible, and thus diminish vulnerability to predators, increase survivorship and the probability to reach reproductive maturity.

## Data Availability

The data that support the findings of this study are available from the corresponding author, Irene Fernández-Rodríguez, upon reasonable request.

## Declarations

### Statement for highlighted student paper

This study gives insight into the evolution of allocation trade-offs that may occur during regeneration. It sheds light on the on the interpretations of previous studies that addressed this question.
